# Comparison of the 3D-Microstructure Between Alveolar and Iliac Bone for Enhanced Bioinspired Bone Graft Substitutes

**DOI:** 10.3389/fbioe.2022.862395

**Published:** 2022-06-17

**Authors:** Rene Rothweiler, Christian Gross, Emely Bortel, Sarah Früh, Javier Gerber, Elodie Boller, Jonas Wüster, Andres Stricker, Tobias Fretwurst, Gerhard Iglhaut, Susanne Nahles, Rainer Schmelzeisen, Bernhard Hesse, Katja Nelson

**Affiliations:** ^1^ Department of Oral- and Craniomaxillofacial Surgery, Faculty of Medicine, Medical Center, University of Freiburg, Freiburg, Germany; ^2^ Xploraytion GmbH, Berlin, Germany; ^3^ European Synchrotron Radiation Facility, Grenoble, France; ^4^ Department of Oral and Maxillofacial Surgery, Berlin Institute of Health, Charité—Universitätsmedizin Berlin, Corporate Member of Freie Universität Berlin, Humboldt-Universität zu Berlin, Berlin, Germany

**Keywords:** bone microarchitecture, osteocytes, morphometry, permeability tensor, synchrotron μ-CT

## Abstract

In oral- and maxillofacial bone augmentation surgery, non-vascularized grafts from the iliac crest demonstrate better clinical performance than alveolar bone grafts. The underlying mechanisms are not fully understood but are essential for the enhancement of bone regeneration scaffolds. Synchrotron Radiation µ-CT at a pixel size of 2.3 μm was used to characterize the gross morphology and the vascular and osteocyte lacuna porosity of patient-matched iliac crest/alveolar bone samples. The results suggest a difference in the spatial distribution of the vascular pore system. Fluid simulations reveal the permeability tensor to be more homogeneous in the iliac crest, indicating a more unidirectional fluid flow in alveolar bone. The average distance between bone mineral and the closest vessel pore boundary was found to be higher in alveolar bone. At the same time, osteocyte lacunae density is higher in alveolar bone, potentially compensating for the longer average distance between the bone mineral and vessel pores. The present study comprehensively quantified and compared the 3D microarchitecture of intraindividual human alveolar and iliac bone. The identified difference in pore network architecture may allow a bone graft from the iliac crest to exhibit higher regeneration potential due to an increased capacity to connect with the surrounding pore network of the residual bone. The results may contribute to understanding the difference in clinical performance when used as bone grafts and are essential for optimization of future scaffold materials.

## 1 Introduction

Bone grafting is a frequently performed intervention in oral and maxillofacial surgery. For this procedure, synthetic, allogenic, and xenogenic bone substitutes and autologous bone grafts are available. Autologous bone remains the gold standard (Galindo-Moreno et al., 2008; Pistilli et al., 2014; Fretwurst et al., 2015a; Sakkas et al., 2017). Depending on the size and quality of the graft required, autologous bone can be obtained from different intra- and extraoral sites, such as the alveolar bone (AB) or the iliac crest (IC) (Nkenke and Neukam, 2014; Kamal et al., 2018). In small-volume augmentations (below 5 mm), bone from the alveolar bone (e.g., ramus mandibulae) has been demonstrated to be successful (Nkenke and Neukam, 2014; Spin-Neto et al., 2014; Khoury and Hanser, 2015; Chappuis et al., 2017; Sakkas et al., 2017). If greater bone volumes are needed, bone from the IC has proven clinically superior (Nelson et al., 2006; Heberer et al., 2009; Fretwurst et al., 2015a; Fretwurst et al., 2015b; Troeltzsch et al., 2016). The reason for the higher competence of the graft from the iliac crest in large-volume augmentations has not been elucidated to date. *In vitro* studies using primary human osteoblasts and bone marrow stromal cells from patient-matched alveolar and iliac bone have demonstrated site-specific phenotypic and functional differences regarding their osteogenic potential, proliferation properties, and gene expression profiles (Akintoye et al., 2006; Akintoye et al., 2008; Wein et al., 2015). Current proteome-based analysis of patient-matched human alveolar and iliac bones emphasizes site-specific differences, with iliac bone predominantly expressing immune-related proteins and alveolar bone ECM-related structural proteins (Fretwurst et al., 2022). This difference in the proteome fingerprint allows for the first insights into the site- and patient-specific nanostructural differences of IC and AB transplants.

The orchestration of intraoral bone graft regeneration depends on the surrounding microenvironment, among which the stable fixation of the graft to the residual bone is imperative as extravasation, diapedesis, and angiogenesis are initiated predominantly from the residual bone (Heberer et al., 2009; Carrel et al., 2016). A further component of successful graft regeneration is the competence of the bone graft or the properties of the scaffold, which is currently defined by its osteogenic, osteoinductive, and osteoconductive properties (Dimitriou et al., 2011). Osteoconductivity describes the capability of passively guiding the ingrowth of vessels and cells of the residual bone provided through a scaffold with a promoting 3D microarchitecture for bone formation during regeneration. This process largely depends on the physical properties of the scaffold, among which wide-open pore structures have been shown to allow higher and faster bone ingrowth (Whang et al., 1999; Karageorgiou and Kaplan, 2005; Feng et al., 2011; Bohner et al., 2017; Weber, 2019; Ghayor et al., 2021). One potential approach is to mimic the structures of the cancellous and cortical bone (Sheikh et al., 2016; Zhang et al., 2020; Qu et al., 2021).

To understand the osteoconductive competence of a bone transplant, its anatomy should be considered, in particular the microarchitecture (e.g., porosity) in the cortical bone and the trabecular structure of the cancellous bone (Fratzl and Weinkamer, 2007; Cooper et al., 2011; Palacio-Mancheno et al., 2014; Bach-Gansmo et al., 2016; Maggiano et al., 2016; Le et al., 2017). Cortical porosity comprises the vascular porosity with its intracortical pore system (IPS) and the osteocyte lacunar–canalicular (LCN) porosity and is considered to be a complex, dynamic biologic network (Marenzana and Arnett, 2013; Cowin and Cardoso, 2015; Maggiano et al., 2016; Grüneboom et al., 2019). The focus has shifted from designing the ideal porosity for osteogenic cell ingrowth to the fabrication of more complex structures of bone scaffolds for multicellular delivery and complex morphologies (Zhang et al., 2020). Only few studies have been published describing the spatial arrangement of the IPS in human long bone and the calvaria. However, a comparative analysis of the spatial arrangement of the IPS considering different human bone entities is still lacking (Cooper et al., 2011; Lafage-Proust et al., 2015; Maggiano et al., 2016; Filipowska et al., 2017; Chen et al., 2020). Studies in mouse bone have revealed an intricate vascular system with interconnected vessels of varying diameter, specific orientation, and function in long bone (Reismann et al., 2017; Grüneboom et al., 2019). The importance of blood flow in bone homeostasis, healing, and regeneration has been recognized by commencing research on proangiogenic strategies in bone tissue engineering (Winkler et al., 2018; Yan et al., 2019; Chen et al., 2020; Gonçalves et al., 2021). Intercommunicating osteocytes residing in lacunae in close proximity to the IPS are considered regulators of bone homeostasis and mechanoreception (Bonewald, 2011; Hemmatian et al., 2017; Repp et al., 2017). The size and arrangement of osteocyte lacunae in osteons was measured in great detail in the femoral bone (Hannah et al., 2010; Carter et al., 2013; Dong et al., 2014; Portier et al., 2020). Data considering the osteocyte lacunae properties in different human bone entities suggest an interindividual, site-specific, and local regional heterogeneity in osteocyte lacunae properties (Klein-Nulend et al., 2013; Hesse et al., 2014a; Hesse et al., 2014b; Hesse et al., 2015; Bach-Gansmo et al., 2016; Hunter and Agnew, 2016; Portier et al., 2020).

The visualization, quantification, and consecutive understanding of the skeletal site-specific 3D microarchitecture may be fundamental for further understanding of the competence of bone transplants and optimizing future scaffold materials. 2D histomorphometric approaches to analyze bone microarchitecture have successfully been utilized, allowing resolutions in the nanometer range when using electron microscopy (Grüneboom et al., 2019; Portier et al., 2020). Synchrotron radiation micro-CT (SR µ-CT) provides an advanced method for non-destructive, high-resolution 3D imaging of bone microarchitecture (Peyrin et al., 2000; Hesse et al., 2014b; Grüneboom et al., 2019; Portier et al., 2020). SR µ-CT uses monochromatic or near-monochromatic beams to produce images with excellent brilliance, signal-to-noise ratio, spatial resolution, and contrast (Hesse et al., 2014b; Grüneboom et al., 2019; Portier et al., 2020). The description and quantification of the bone microarchitecture today are often based on a standardized nomenclature, as described by the American Society for Bone and Mineral Research (Dempster et al., 2013). The defined parameters are based primarily on observations from two-dimensional histomorphometry. However, their volumetric modifications were adapted for 3D methods, especially from the perspective of understanding the 3D complexity of the bone (Bouxsein et al., 2010). In 1989, Feldkamp et al. (1989) first published a method adapting conventional histomorphometric parameters to µ-CT scans. Parameters such as bone volume fraction (BV/TV), porosity (1−BV/TV), specific bone surface (SA/BV), trabecular thickness (Tb.Th), and trabecular separation (Tb.Sp) have become standard parameters for the morphometric analysis of synchrotron µ-CT scans of bone samples. Synchrotron µ-CT enables the differentiation of the vascular and LCN porosity by using both the phase contrast enhancement mode and quantitative phase retrieval (Langer et al., 2012; Wolfram et al., 2016; Gauthier et al., 2019; Portier et al., 2020). The spatial arrangement of the IPS and osteocyte lacunae is not routinely assessed as it still remains a challenge. The spatial orientation, however, seems essential to understand the substance transport dynamics in bone and bone graft regeneration (McCarthy, 2006; Carrel et al., 2016). The IPS is a fluid-filled structure with its dynamics depending on the size, orientation, and course/interconnection (Cowin and Cardoso, 2015; Cavo and Scaglione, 2016; Ramasamy et al., 2016; van Tol et al., 2020).

This study aimed to analyze and compare the microarchitecture in patient-matched alveolar and iliac bone on a microscale using synchrotron computed tomography. The spatial arrangement and the differences in permeability of the IPS between AB and IC were quantified using computational techniques based on the acquired 3D-image data.

## 2 Materials and Methods

The study has been approved by the local ethics committee of the Albert-Ludwigs-Universität Freiburg, Germany (EK 603/15). This study was performed in accordance with the Helsinki Declaration of 1964, as revised in 2013.

### 2.1 Patient Selection and Sample Harvesting

Ten healthy patients (five females and five males; mean age: 56 years; range: 46–67 years), scheduled for alveolar bone grafting with autologous, non-vascularized anterior superior iliac crest (IC) bone block, were included in the study after providing oral and written consent. All participants were treated at the Department of Oral and Maxillofacial Surgery of the University Medical Center Freiburg between 2017 and 2018, meeting the inclusion criteria: healthy patients with no systemic disease (e.g., osteoporosis and cancer), no regular medication affecting bone homeostasis (e.g., antiresorptive agents), and tooth extraction more than 6 months prior to grafting. For each patient, a biopsy sample from the recipient site [edentulous region of the alveolar bone (AB) (*N* = 10)] and the donor site [anterior superior iliac crest (IC) (*N* = 10)] was retrieved using either a saw or a chisel during contouring of the graft or the recipient graft site. In the course of graft conditioning, IC biopsies were retrieved either from the right or the left IC following a standardized surgical protocol (Nelson et al., 2006; Heberer et al., 2009; Fretwurst et al., 2015c). Depending on the site of augmentation, the AB biopsies originated either from the maxilla or the mandible and were retrieved from the cortical portion of the jaw in the course of recipient site preparation. Detailed patient and sample data are listed in Table 1.

**TABLE 1:** Patient and sample data.

Patient index	Gender	Age (years)	Biopsy index	Biopsy origin
P1	Female	67	P1 (AB)	Maxilla (region 11)
			P1 (IC)	Anterior superior iliac crest
P2	Male	67	P2 (AB)	Mandibulae (region 46)
			P2 (IC)	Anterior superior iliac crest
P3	Male	48	P3 (AB)	Mandibulae (region 45)
			P3 (IC)	Anterior superior iliac crest
P4	Male	54	P4 (AB)	Mandibulae (region 41)
			P4 (IC)	Anterior superior iliac crest
P5	Female	56	P5 (AB)	Maxilla (region 11)
			P5 (IC)	Anterior superior iliac crest
P6	Male	46	P6 (AB)	Maxilla (region 11)
			P6 (IC)	Anterior superior iliac crest
P7	Female	52	P7 (AB)	Maxilla (region 11)
			P7 (IC)	Anterior superior iliac crest
P8	Male	52	P8 (AB)	Mandibulae (region 46)
			P8 (IC)	Anterior superior iliac crest
P9	Female	65	P9 (AB)	Mandibulae (region 45)
			P9 (IC)	Anterior superior iliac crest
P10	Female	53	P10 (AB)	Maxilla (region 11)
			P10 (IC)	Anterior superior iliac crest
N = 10	50% female; 50% male	Ø 56 SD: 7.69	N = 20	Recipient site: 50% maxilla, 50% mandibular, Donor site: 100% anterior superior iliac crest
Age is given as the mean and standard deviation (SD)				

### 2.2 Sample Preparation

Bone specimens were fixed in 4% neutral buffered formalin after harvesting. The specimens were subsequently dehydrated in an ascending alcohol series (water, 70%/80%/96%/100% ethanol) for 3 days each, defatted in xylene and infiltrated, embedded, and polymerized in Technovit^®^ 9100 (Heraeus Kulzer, Wehrheim, Germany), a polymethyl methacrylate (PMMA)-based technical resin, according to the manufacturer’s instructions and as described previously (Oshima et al., 2014). The Technovit^®^ blocks were then cut to a size of about 5 mm × 5 mm using a band saw (Proxxon S.A., Wecker, Luxembourg).

### 2.3 Synchrotron Radiation Micro-CT

µ-CT data were acquired at the beamline ID 19 of the European Synchrotron Radiation Facility (ESRF; Grenoble, France) using a (pink-) beam energy of 46.9 keV and a sample detector distance of 460 mm. A total of 4,000 radiographs over an angle of 360° were captured, and the acquisition time per frame was 20 ms. The detector comprised 2,560 × 2,160 pixels and an optical system resulting in an effective pixel size of 2.27 μm (camera: pco.edge 5.5, PCO AG, Kelheim, Germany). Reconstruction was performed using Paganin’s method in combination with the conventional filtered back projection algorithm, applying a delta/beta ratio of 350 (Paganin et al., 2002). Reconstructed data were stored in units of refractive indices in units of 2π/λ, with *λ* being the wavelength of the X-ray beam, referred to as gray value data, stored in 32 bit floating values.

### 2.4 Digital Bone Morphometry and Osteocyte Lacunae Segmentation

**FIGURE 1:**
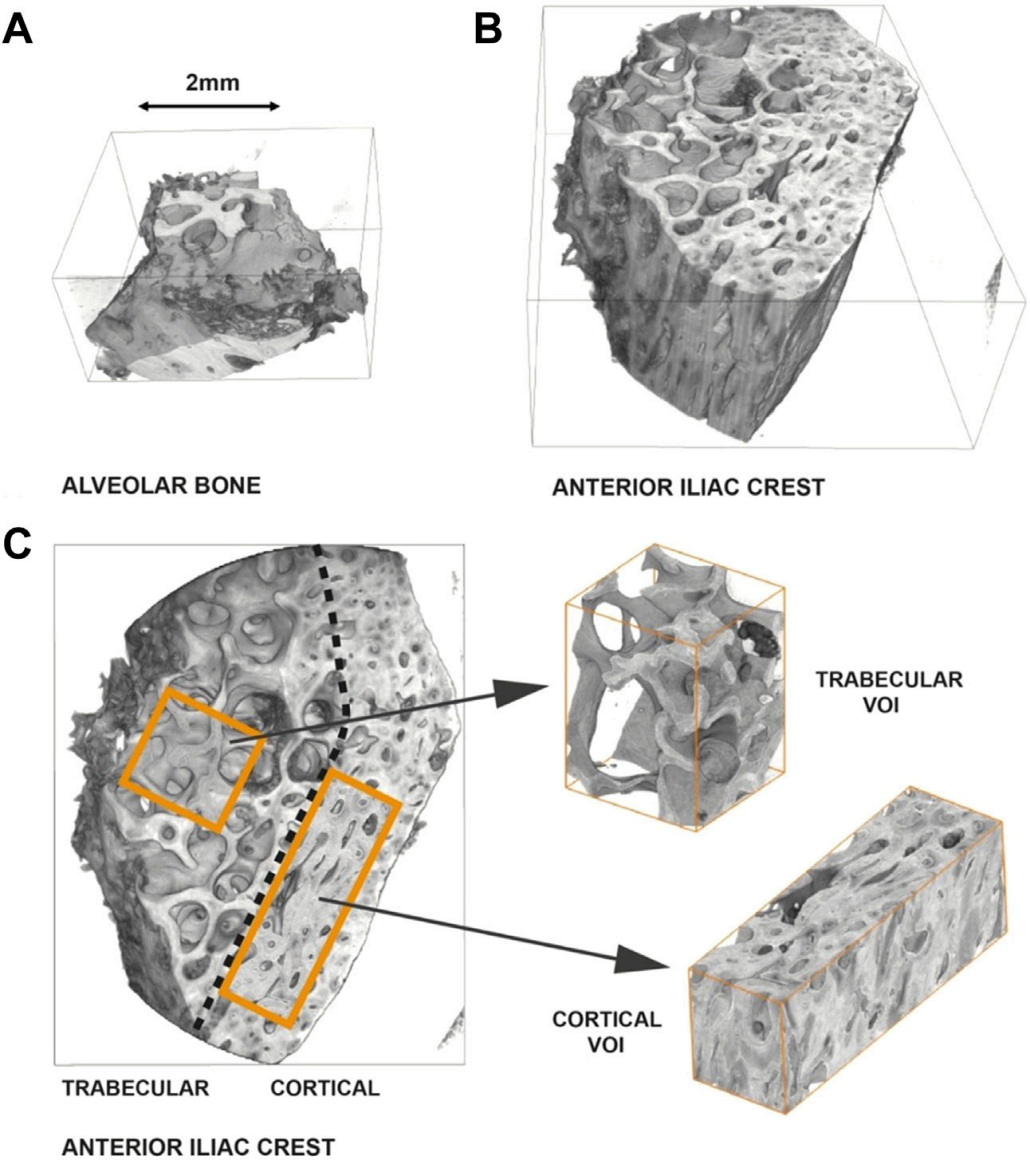
Representative reconstruction and selection of volumes of interest–Sample P4 (AB)/(IC). **(A)** 3D-rendering of an alveolar bone sample. **(B)** 3D-rendering of an iliac crest sample. The IC sample is greater in size than the AB sample. Furthermore, the AB primarily consists of cortical bone, as opposed to the IC sample, which is clearly composed of a trabecular and a cortical region. **(C)** Differentiation of the IC sample into a cortical and a trabecular region and definition of volumes of interest (VOIs).

Representative, individual-sized volumes of interest (VOIs) were selected within the AB, cancellous iliac crest [IC (CA)], and cortical iliac crest [IC (CO)] samples. The VOIs have been selected to include artifact-free (e.g., free of motion artefacts) and undamaged bone anatomy (e.g., cracks induced by sample preparation or handling). Figure 1 displays an exemplary reconstructed 3D rendering of an AB (A) and an IC (B) sample. A classification as to cortical or cancellous bone was applied for the IC bone. AB was by definition allocated to be cortical bone tissue.

The following parameters of bone morphometry were investigated: 1) bone volume (BV), 2) total volume (TV), 3) bone surface area (SA), 4) bone volume fraction (BV/TV), 5) porosity (1−BV/TV), 6) specific bone surface (SA/BV), and 7) osteocyte lacunae density (N.Lc/BV). For digital bone morphometry, an adapted segmentation workflow was developed using ImageJ [distribution FIJI (Schindelin et al., 2012)], MorpholibJ plugin (Legland et al., 2016), and MATLAB (R2018b, The Mathwork Inc., Natick, MA, United States).

#### 2.4.1 Image Segmentation and Image Analysis

After identifying VOIs, each volume was segmented into its different regions, namely, mineralized tissue, vessel pores (Haversian canal or Volksmann’s canal), extratrabecular space, osteocyte lacunae, and regions of the volume not belonging to the sample (surrounding air and sample holder).

A canal mask (void spaces) was generated by applying a 3D gray scale attribute closure with 151 voxels followed by a 3D median filtering with 6 voxels. With the subsequent thresholding and a dilation of 1 voxel, the canal voxels were set to a value of 1. To obtain the masks for the bone regions, a 3D median filter with a value of 6 voxels was applied to the gray value data.

Osteocyte lacunae were extracted through a bottom-hat transformation coupled to subsequent thresholding of the transformed image. A connected component analysis was applied to size-filter the osteocyte lacunae (criteria were that lacunae be >5 and <150 voxels; see Supplementary Figure SA1 for an exemplary representation of the lacunae within a bone sample).

From the obtained masks, the BV was calculated as the number of voxels allocated to mineralized bone (plus the voxels allocated to osteocyte lacunae), and TV is the number of voxels allocated to pores and mineralized tissue.

#### 2.4.2 Arrangement of the Intracortical Pore System

To investigate the arrangement and orientation of the IPS, VOIs within the examined samples had to fulfill several criteria: 1) set within the cortical bone, 2) not containing any voids not belonging to the cortical canal system (i.e., cracks, space outside the sample), 3) still being a sufficiently large, representative volume. Three sample pairs {P4 (AB)/[IC(CO)], P8 (AB)/[IC(CO)], and P9 (AB)/[IC(CO)]} contained VOIs fulfilling these requirements and were considered eligible for the analysis of the IPS. The selected VOIs were, thus, different and smaller than the VOIs used for the determination of bone morphogenetic parameters and osteocyte lacunar density determination.

The spatial arrangement of the canals within the bone matrix was quantified by analysis of the canal and BV mask’s distance map. To quantify the distance of mineralized tissue to a pore interface (excluding lacunar porosity), a 3D Euclidean distance transform was performed on the mask of the mineralized tissue, called BV _dist_. A mask of the image boundaries and non-tissue region was eroded by 50 pixels and multiplied with BV _dist_ to minimize image boundary caused artifacts. The result was then used to compute the histogram of the distance values and generate the cumulative sum. The values at which 50% and 95% of the mineralized tissue are located with respect to the nearest pore were computed. The same was carried out for the canal volume with respect to the BV boundary (Hesse et al., 2014a).

**FIGURE 2:**
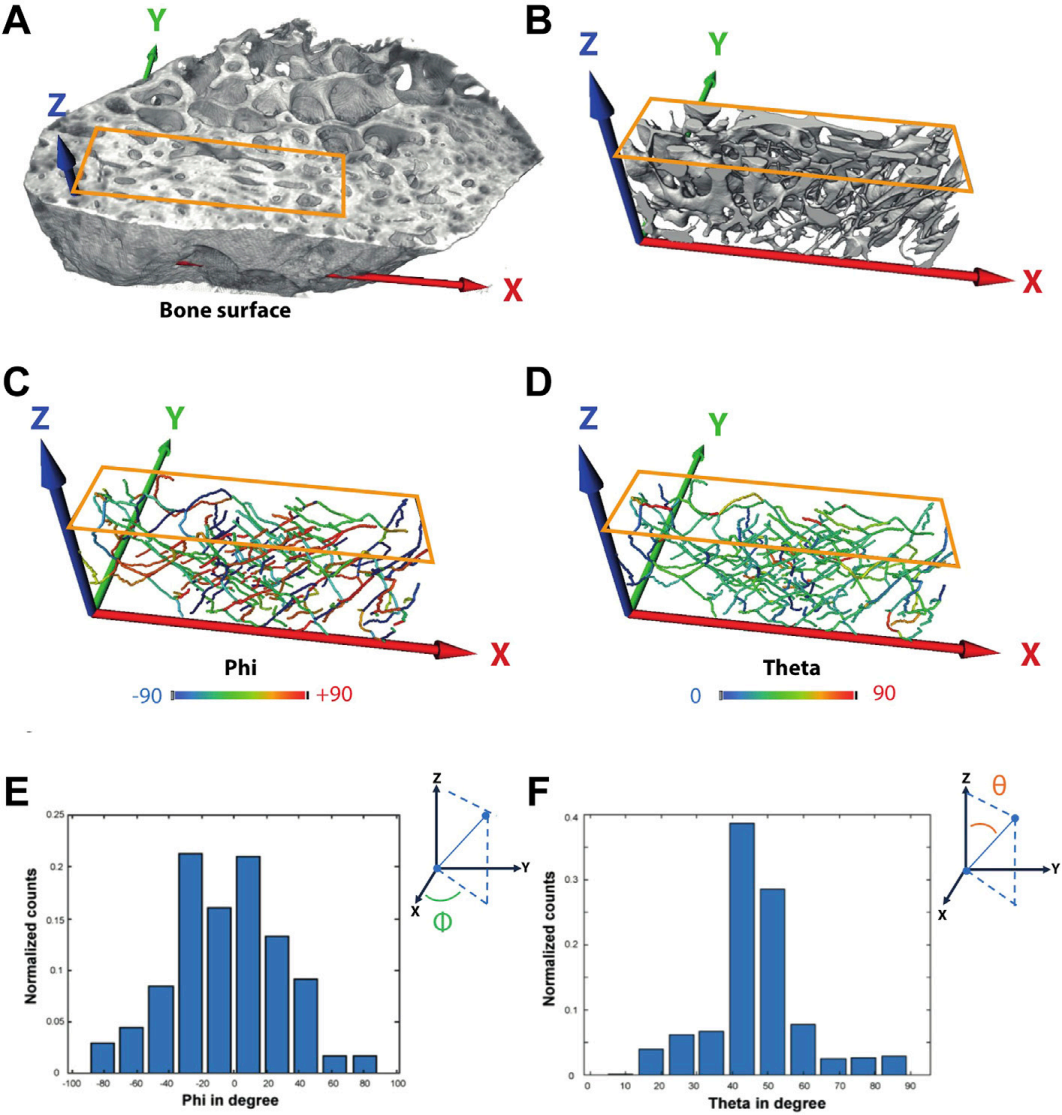
Exemplary definition of the axis and calculation of angles phi and theta—Sample P4 (IC). **(A)** Volume rendering of the full sample indicating the region used for further analysis of the vessel orientation. **(B)** Surface renderings of the intracortical canal system. **(C–F)** Angles phi and theta were computed for each segment afterward.

For IPS orientation analysis, the investigated VOIs were aligned in such a way that the anatomical long axis was parallel to the z-axis of the global coordinate system and the bone surface within the x–z plane. Skeletons (also called centerlines) of the extracted canals were computed using Thermo Scientific Avizo Software (v. 2019.3, Thermo Fischer Science Inc., MA, United States). The Euclidean distance to the closest surface is stored at each point of the skeleton. This is used to estimate the average pore thickness. From each segment of each skeleton, the angles theta and phi have been computed in order to quantify the spatial orientation of the canal system. Theta, the angle with respect to the long axis (z), can be between 0° and 90°. In the plane perpendicular to z (x, y plane), phi can have values from −90° to 90°. An angle of 0° indicates that the segment is parallel to the x-axis and thus to the bone surface (Figure 2). Histograms of phi and theta have been calculated after weighting each segment by its length. Subsequently, in these histograms, the variance of the bin height has been calculated for both phi and theta. The variance of the bin size is high when there are pronounced peaks in these histograms. Contrarily, if all angles were randomly distributed, there would be no peak in the histogram, and thus the variance would be the smallest. Since the angles are interrelated, we define the product of the variances as a descriptor for the alignment, called the level of alignment (LOA) (Bortel et al., 2022).

### 2.5 Fluid Simulation

The regions (VOIs) used for the analysis of the IPS orientation by analyzing the skeletons (described in Section 2.4) were further used as input for a fluid simulation. In addition to the calculation of the absolute permeability *via* the Absolute Permeability Experiment Simulation module of Avizo software, the tensor of the absolute permeability was calculated *via* the Absolute Permeability Tensor Calculation module (Thermo Scientific Avizo Software, XLab Module, Version 2020.2).

Absolute permeability is defined as the measure of the ability of a porous material to transmit a single-phase liquid in units of darcy (d), with 1d = 0.987 μm^2^. With λ_1_, λ_2_ and λ_3_ being the permeability along X, Y and Z, respectively. Through Darcy’s law, the fluid and flow were computed. To numerically estimate the absolute permeability, the Stokes equations are applied (Bortel et al., 2022).

This permeability tensor provides additional information about the intensity of permeability along any spatial direction. From it, the anisotropy of a porous medium can be derived, for example, the dependence of the permeability intensity on the flow direction. The result of the tensor calculation is the absolute permeability tensor, which indicates the permeability in all three spatial directions and the eigenvectors and the associated eigenvalues.

The ratio of the eigenvalues to each other was then determined to quantify the anisotropic behavior of the absolute permeability.

The termination conditions for the simulations were that either the convergence criterion was smaller than 10^−5^ or the number of iterations reached 10^6^. To quantify the anisotropy of the permeability, the mean of the ratios of E1/E2 and E1/E3 was computed, with E1, E2, and E3 being the eigenvalues of the permeability matrix.

### 2.6 Generating Surfaces of the 3D Geometry

The abovementioned VOIs were converted to surfaces and exported as .stl files. These will be made available and can be used for 3D printing. Avizo was used to generate a surface out of the binary bone data. Binning the data to reduce the size of the surface and smoothing led to the loss of small canals (Supplementary Figure SA2) such that the unbinned data were converted with a mild smoothing (kernel 1 voxel), resulting in surfaces containing several millions of triangles. To reduce the file size but to keep the structure as precise as possible, the surface was simplified down to 1 million triangles. Remeshing with a smoothing threshold of 0.6 led to a final smoothed surface with preserved hard edges containing 500 k uniformed triangles (Supplementary Figure SA3).

### 2.7 Statistics

Normal distribution was tested graphically and statistically (Shapiro–Wilk test). Descriptive/explorative statistics and statistical hypothesis tests (ANOVA, Kruskal–Wallis test, Mann–Whitney test, *t*-test) with post-hoc testing (Bonferroni) were performed using IBM SPSS Statistics (version 25.0, released in 2017, IBM Corp., Armonk, NY, United States). *p*-values ≤ 0.05 were considered statistically significant. No statistical analyses were performed for the arrangement of the IPS investigation due to the limited number of samples investigated (*n* = 3 pairs).

## 3 Results

### 3.1 Comparison of Bone Morphometric Parameters

The data set for the investigated bone morphometric parameters is presented in Supplementary Table SA1. The size of the biopsies retrieved (TV) varied due to ethical and surgical considerations of only using bone specimens acquired by bone conditioning, resulting in the TV of AB being smaller.

**FIGURE 3:**
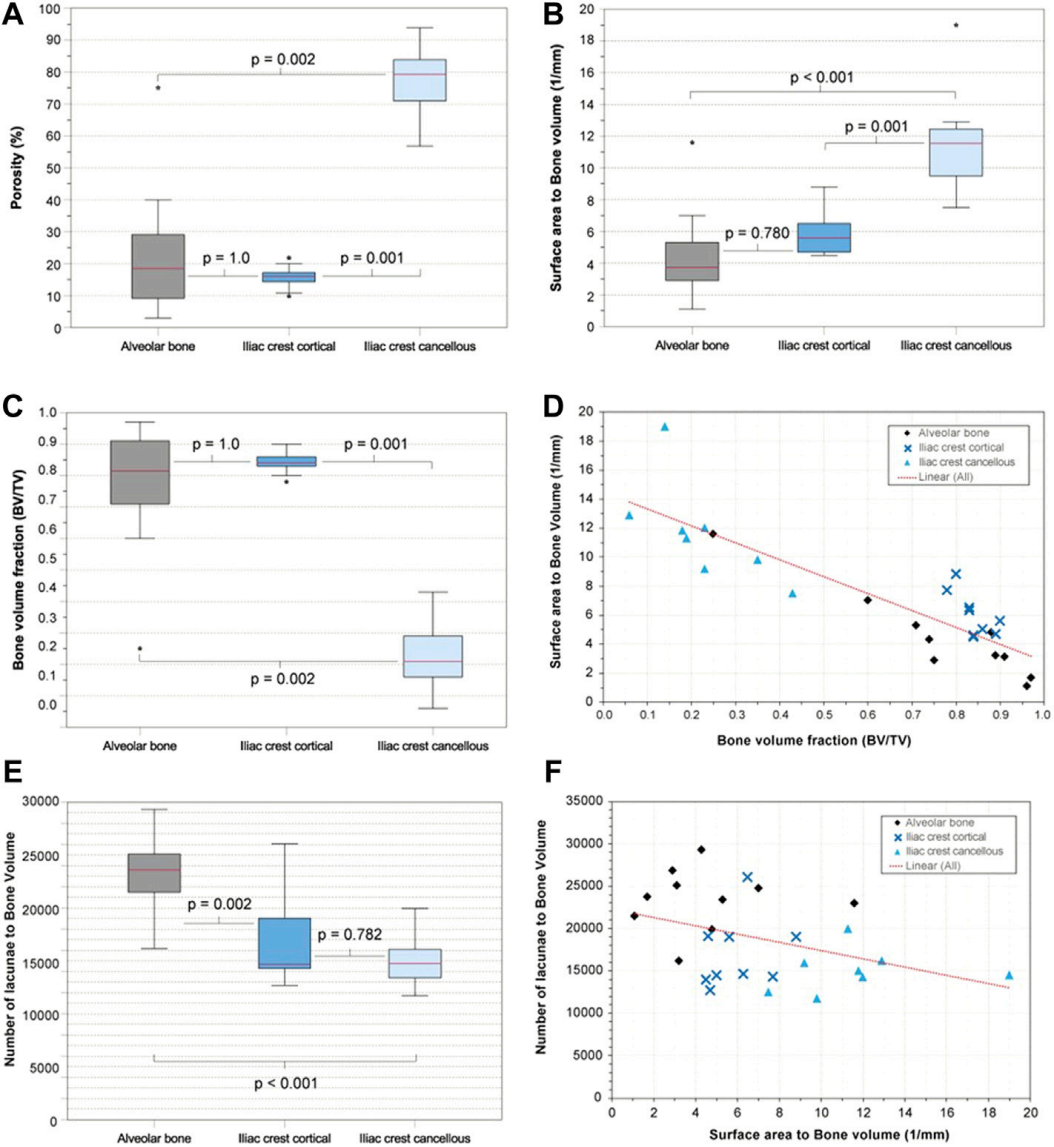
Alveolar bone vs. anterior iliac crest—selected parameters. **(A)** Boxplots visualizing porosity (%) of the examined bone types. **(B)** Surface area (SA) to bone volume (BV) ratio (SA/BV; 1/mm) of the examined bone types as boxplots. **(C)** Bone volume fraction (BV/TV) of the examined bone types as boxplots. **(D)** Scatterplot with SA/BV on the y-axis and bone volume fraction (BV/TV) on the x-axis **(E)** Osteocyte lacunar density of the examined bone types as boxplots. **(F)** Scatterplot with the lacunar density on the y-axis and SA/BV on the x-axis.

The interskeletal site comparison of the derived bone parameters BV/TV, porosity, and SA/BV based on the larger volume VOIs in 10 samples showed apparent similarities between AB and IC (CO). There were no significant differences between AB and IC (CO) concerning BV/TV, porosity, and SA/BV, whereas the interindividual variation of the values was higher in AB and IC (CA) than in IC (CO). AB and IC (CO), however, differed significantly from IC (CA) in all parameters (*p*-values given in Figures 3A–C), highlighting the microarchitectural differentiation of trabecular and cortical bone.

### 3.2 Comparison of Osteocyte Lacunae Density

The resolution and contrast allowed the segmentation of the osteocyte lacunae in all samples. The total number of osteocyte lacunae analyzed in the study population was 1.6 × 10^6^. The osteocyte lacunae density (N.Lc/BV) of AB (23,389 + −3,659), IC (CO) (17,029 + −4,209), and IC (CA) (15,028 + −2,525) revealed that AB features significantly more lacunae than IC (CO) (*p* = 0.002) and IC (CA) (*p* < 0.001). There was no significant difference between IC (CA) and IC (CO) (Figures 3E,F).

### 3.3 Analysis of the Intracortical Pore System Geometry

**FIGURE 4:**
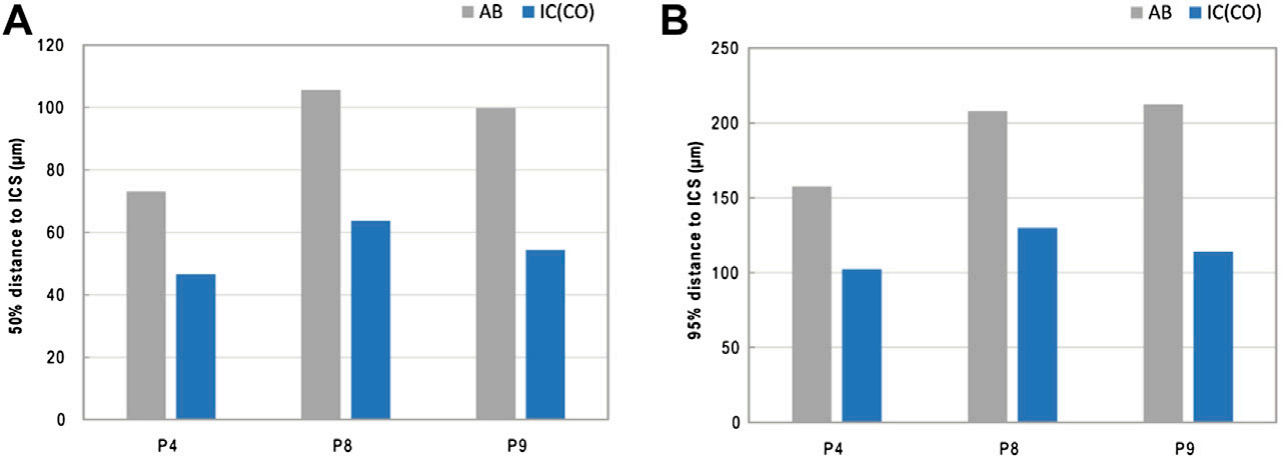
IPS arrangement—distance of mineralized bone to the intracortical surface ICS. **(A,B)** Graphs showing the average 50% **(A)** and 95% **(B)** distance of the mineralized bone to the closest ICS.

Arrangement analysis of the IPS revealed that the average distance of the mineralized bone phase in IC tends to be closer to the IPS than in AB. On average, 50% of the mineralized bone tissue was found to be within a distance of ∼55 µm (IC) and ∼93 µm (AB) to the nearest canal surface. On average, 95% of mineralized bone was found to be within ∼115 µm (IC) and ∼193 µm (AB) of the canal surface. The average pore size was slightly higher in IC (∼59 µm) than in AB (∼51 µm) (Figure 4; Table 2; Supplementary Figure SA4).

**TABLE 2:** Parameters describing the arrangement of the intracortical pore system (IPS).

Sample ID	IPS surface to total volume (1/mm)	IPS surface to bone volume (1/mm)	IPS porosity (%)	IPS mean pore size (µm)	Dist. mineral 50% (µm)	Dist. mineral 95% (µm)	Dist. IPS 50% (µm)	Dist. IPS 95% (µm)	Phi bin var (E)	Theta bin var (E)	Level of alignment (var bin phi * var bin theta)	Level of alignment ratio (IC/AB)
P4 (AB)	3.0	3.5	16	59.2	73.1	157.5	23.8	99.2	4.4–3	4.4–3	1.9E-05	4.9
P4 [IC (CO)]	5.6	6.9	24	56.2	46.5	102.4	18.6	66.5	5.7–3	16.5–3	9.5E-05	
P8 (AB)	1.4	1.4	2	43.4	105.6	207.7	7.3	22.2	5.0–3	6.2–3	3.1E-05	1.7
P8 [IC (CO)]	3.1	3.2	5	47.0	63.6	129.8	7.9	30.6	5.0–3	10.7–3	5.3E-05	
P9 (AB)	2.3	2.5	6	49.6	99.7	212.2	14.3	64.7	2.3–3	7.4–3	1.7E-05	9.0
P9 [IC (CO)]	6.1	7.5	23	75.1	54.3	114.0	15.9	49.7	5.8–3	27.0–3	15.6E-05	

**FIGURE 5:**
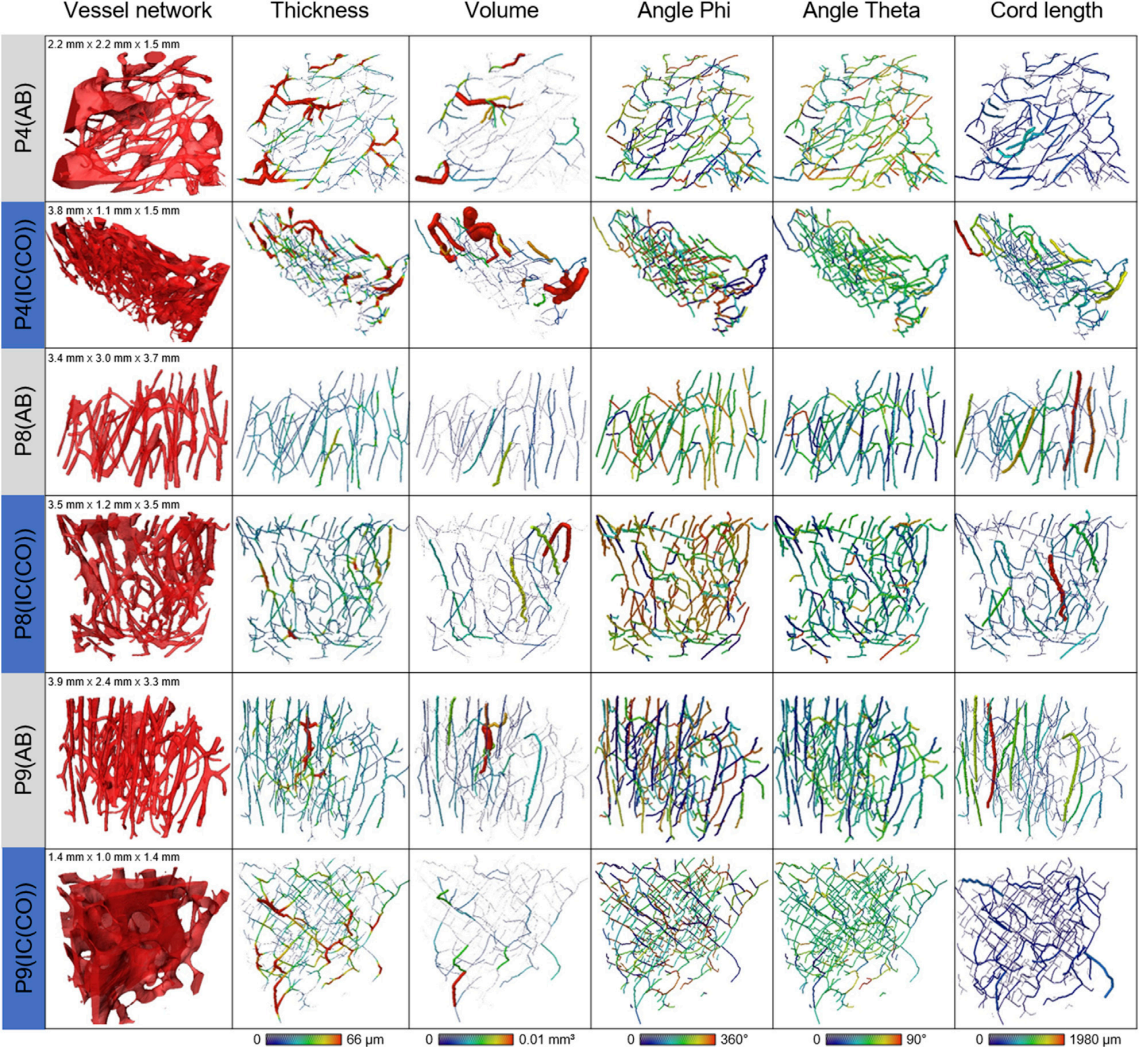
Summary of the skeletonized vessel pores comparing the volumes, thickness, and orientation of the skeleton fragments between AB and IC (CO). Visualizations of **(A)** vessel network, **(B)** thickness, **(C)** volume, **(D)** angle phi, **(E)** angle theta, and **(F)** cord length.

The results for the spatial orientation of the IPS are summarized in Table 2. For all the investigated samples, the level of alignment (LOA) was higher for the IC than for AB. This is emphasized by the observation that the ratio LOA IC/AB was above the value of 1 for all three investigated pairs of samples. The alignment of the IPS showed a higher orientation in bone from IC in comparison to AB, exemplarily shown in Figure 5.

### 3.4 Simulation of Fluid Dynamics

**FIGURE 6:**
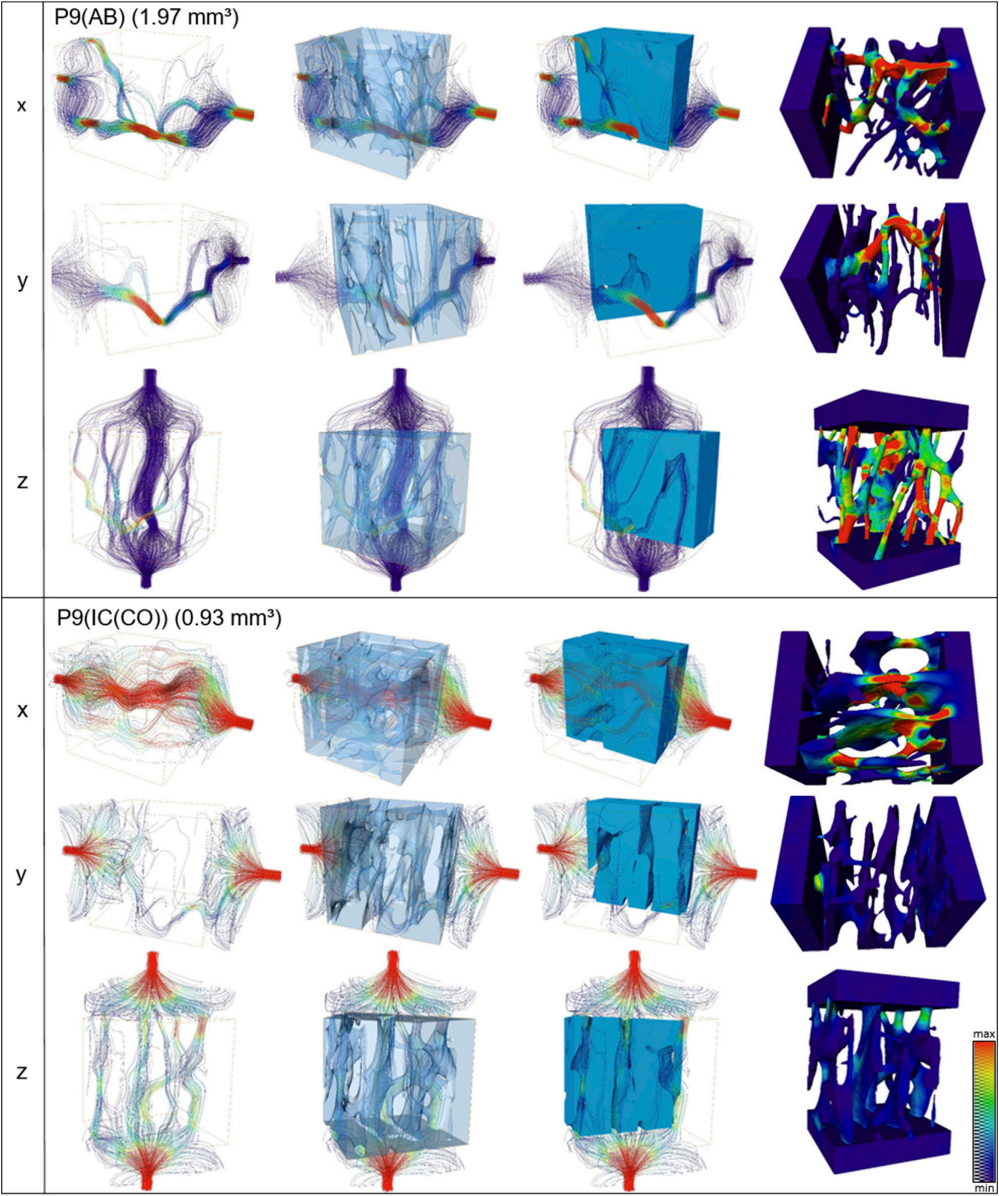
Streamline (first three columns) and velocities (last column) of fluid simulations performed along x, y, and z axes for alveolar and iliac crest of one patient (P9). The absolute permeability values of this patient are shown in Table 3.

Fluid simulations on cubes virtually cut from the volumes discussed in the previous section reveal that the permeability along the z-axis is the highest for four out of six analyzed samples. The mean permeabilities of all three IC samples are higher than those of the two AB samples (Figure 6). The sizes of the cubes are set such that they contain as much bone tissue as possible and therefore differ in size. However, the differences in mean permeabilities cannot be explained by cube size alone. To quantify the anisotropy of the permeability tensor, the eigenvalues of this matrix were computed and the means of E2/E1 and E3/E1 were calculated. Anisotropy of the permeability is more pronounced in AB than in IC. Thus, not only is the average permeability higher in the analyzed regions in IC as compared to AB but also the permeability anisotropy is less pronounced in those samples. The sample shape and size of sample P4 (AB) did not allow extracting a cube of a representative volume. Eigenvalues (with E1>E2>E3) of the permeability tensor and their ratios are also computed. The smaller the ratios (E2/E1 or E3/E1), the more pronounced the impact of fluid direction on the permeability (Table 3).

**TABLE 3:** Summary of the fluid simulations quantifying the direction-dependent permeability of the different bone specimens.

	From experiment							From tensor			
Sample	λ_1_	λ_2_	λ_3_	#cubes	Dimensions	TV in mm³	(λ_1_ + λ_2_)/(2λ3)	TV in mm³	EV2/EV1	EV3/E1	(E2 + E3)/(2E1)
P4 (AB)	—	—	—	0	—		NaN	3.2	0.11	0.02	0.06
P4 [IC(CO)]	1.47	0.01	2.19	3	504³ vxl	1.50	0.34	6.4	0.50	0.12	0.31
P8 (AB)	0.01	0.00	0.15	1	504³ vxl	1.05	0.03	5.4	0.38	0.02	0.20
P8 [IC(CO)]	0.13	0.02	0.43	2	370³ vxl	0.59	0.17	5.2	0.45	0.06	0.26
P9 (AB)	0.25	0.15	1.65	1	552³ vxl	1.97	0.12	9.9	0.32	0.00	0.16
P9 [IC(CO)]	1.64	0.11	1.53	1	430³ vxl	0.93	0.57	1.8	0.57	0.05	0.31

Fluid simulations were performed for virtually cut cubes separately for *X*, *Y,* and *Z* directions and their means and ratios are calculated.

### 3.5 Surface Extraction

The high spatial resolution of the CT scans allowed the exact representation of the vessel pores by a surface mesh (Supplementary Figure SA2A). Binning (x5) of the data shows that a lower resolution leads to a loss of small connections between vessels, such that a precise structure representation is not possible anymore (Supplementary Figure SA2B)*.* The surface geometry of the complex three-dimensional structure of the unbinned bone masks for all six VOIs is represented by triangle meshes (Supplementary Figure SA3).

## 4 Discussion

The present study compared the patient-specific 3D-morphology of alveolar bone and iliac bone to further decipher the difference in graft morphology.

At the time of bone augmentation, patient-matched biopsies were retrieved from the edentulous region of the alveolar bone and the iliac crest and were subjected to synchrotron radiation analysis. In all patients, the removal of the tooth has been performed at least 6 months prior to grafting, ensuring a healed site (Nahles et al., 2013; Atieh et al., 2021). However, minor differences in morphology may have been present due to remodeling processes compared to other regions without prior tooth extraction. Common bone morphometric parameters were evaluated and compared. Macroscopically, bone retrieved from the iliac crest showed a cortical and trabecular structure, whereas the alveolar bone was of cortical nature due to the mode of sample retrieval. The mean porosity of the alveolar bone (AB) was comparable to that of the cortical part of the bone from the anterior superior iliac crest IC (CO) with interindividual variation. This is in accordance with the findings in studies evaluating the morphological changes of the iliac bone in men and women with regard to age (Bach-Gansmo et al., 2016; Recker et al., 2018). This interindividual and intraskeletal variation has been described (Chappard et al., 2013; Hunter and Agnew, 2016).

The investigation of the bone morphometric parameters, including all 10 samples, revealed that the IC (CO) and AB have similar bone morphometric parameters but differed significantly from the IC (CA). In contrast to these results, a μ-CT morphometric comparison by Kamal et al. postulated distinct microarchitectural differences between the human jawbone and IC bone samples (Kamal et al., 2018). However, details have been lost in their study due to the missing differentiation between cortical and cancellous bone compartments in the biopsies retrieved. In general, this differentiation is essential for any morphometric interpretation, but it is not trivial to delineate the cortical bone at the iliac crest as it is located adjacent to the trabecular bone without a strict demarcation (Bach-Gansmo et al., 2016). Within this study, common bone morphometric parameters were evaluated using the larger sized volume of interest (VOI) (VOI corresponded to the maximal possible volume), demonstrating no significant difference in the porosity between IC (CO) and AB. For fluid permeability and vessel-pore network characterization, a refining of the VOI, closer to the outer boundary of the bone (cortices) and smaller in total size, revealed a higher porosity of IC (CO) than that of AB, underlining the complexity of the delineation of the cortical from trabecular bone in humans and hinting at a site-specific difference in the cortical structure. The tendency of a greater mean pore size in IC (CO) might contribute to better osteoconduction as a wide-porous scaffold allows faster bone ingrowth (Weber, 2019; Ghayor et al., 2021). This difference in mean pore size was seen in the small-volume samples. A larger cohort would be necessary to analyze if this becomes a significant finding. Previous studies have shown site-specific differences in osteocyte lacunar density between different bone entities (Hesse et al., 2014a; Hesse et al., 2014b). The osteocyte lacunar density in our study was significantly higher in alveolar bone when compared to the corresponding iliac crest sites. A comparison of the osteocyte density of patient-matched iliac and alveolar bone has not been performed to date. The difference in osteocyte lacunar density between non–patient-matched alveolar bone and tibia and femur has been described, with alveolar bone showing a different number of osteocytes per mm^3^ bone volume and interindividual variation (Hannah et al., 2010; Carter et al., 2013; Hesse et al., 2014a; Hesse et al., 2014b; Carter et al., 2014; Dong et al., 2014; Hesse et al., 2015; Gauthier et al., 2019; Iezzi et al., 2020; Portier et al., 2020). This intersite-specific variation of osteocyte lacunar density has been shown to be associated with a difference in biomechanical strain occurrence and bone turnover-rate (Zimmermann et al., 2011; Iezzi et al., 2020). *In vitro* studies demonstrated a higher proliferation rate of patient-matched primary osteoblasts derived from the alveolar bone when compared to the iliac bone, emphasizing a difference in phenotype (Akintoye et al., 2006; Wein et al., 2015; Wein et al., 2019).

In addition to the difference in osteocyte lacunar density, a difference in the distance of the average mineralized tissue from the pore-vessel boundary was found. In AB, the distance of the mineralized tissue from the next pore-vessel boundary was higher (up to ×1.7) when compared to IC (CO). This is, however, limited by the small sample number that could be included into the analysis (three patients). Nevertheless, these findings might allow the speculation if the higher lacunar density in alveolar bone is compensating for a longer average distance to the next vessel-pore boundary. It is well-accepted that the osteocyte lacunar network strongly contributes to the tissue vascularization and thus to the homeostasis of the mineralized tissue (Mishra and Knothe Tate, 2003; Beno et al., 2006; Cardoso et al., 2013; Weinkamer et al., 2019; van Tol et al., 2020). Bone mineralization heterogeneity is proposed to be the result of remodeling and mineralization processes, which are known to vary across skeletal sites (Lerebours et al., 2020). Previous studies in the human femur revealed a higher degree of mineralization in the human femoral cortex than in the trabecular bone (Buenzli et al., 2018). Initially, this difference in bone mineral density between cortical and trabecular bone was associated with a difference in turnover rate, so an increased average bone mineral density and broader bone mineral density peaks are related to a lower turnover rate, providing more time for secondary mineralization (Roschger et al., 2008; Ruffoni et al., 2008; Buenzli et al., 2018). This hypothesis is debatable as a current computational study, and studies examining human bone subjected to treatment with antiresorptive agents (minimizing bone turnover) reveal no difference in the bone mineral density compared to healthy individuals (Lerebours et al., 2020). This hypothesis is in accordance with the findings of the current study showing a greater distance of mineralized bone to the pore-vessel boundary in the alveolar bone paired with a higher osteocyte lacunar density and suggests alternative mechanisms responsible in addition to bone turnover as the alveolar bone is known to exhibit a high turnover rate (Mavropoulos et al., 2004). A difference in mineralization kinetics due to mechanical requirements and the role of the osteocyte lacuna–canalicular network are currently discussed (Hesse et al., 2014a; Hesse et al., 2014b; Lerebours et al., 2020).

The knowledge of the arrangement of the intracortical pore network is not only important for mineralization kinetics and mechanosensation but also for the dynamics of the transport of biologics involved in graft regeneration (Marenzana and Arnett, 2013; Elsharkawy and Mata, 2018; Hendriks and Ramasamy, 2020; van Tol et al., 2020). The majority of literature evaluating the dynamics of the fluid system is limited to the cortex of long bones (Cardoso et al., 2013; Cowin and Cardoso, 2015). Different levels of bone porosity entertain the bone fluid system, with the vascular porosity being the largest in the lineal dimension (Ciani et al., 2005; Cowin et al., 2009; Cardoso et al., 2013; Cowin and Cardoso, 2015). The measurement of the permeability of the vascular porosity is not trivial due to the intertwining of the vascular pores (van Tol et al., 2020). Anatomic and perfusion studies of long bones using tracers suggest a circulation arranged parallel to the longitudinal running nutrient artery along the surface of the bone (Lopez-Curto et al., 1980; Parfitt, 2000; Fritton and Weinbaum, 2009). The skeletonization and spatial resolution of the IPS revealed a higher level of alignment in AB than in IC (CO). The orientation of the IPS in AB was predominantly along the longitudinal axis (z-axis, parallel to the surface) of the cortex, as described in the human femoral and radial bone (Chappard et al., 2013; Gauthier et al., 2019), whereas in the cortex of the iliac crest, the intracortical vasculature network is less pronounced in one direction but shows a more multidirectional orientation. Furthermore, the higher ratio of the vessel surface to total volume, that is, vessel surface to bone volume results in an average higher overall pore-vessel porosity in the bone from the iliac crest. This might contribute to a higher and more homogenized multidirectional flow of blood and nutrition as a consequence.

Further spatial analysis of the IPS distribution, orientation, and fluid permeability confirmed these findings. Fluid flow simulations also show that the permeability is pronounced in one direction in the alveolar bone (z-axis), while the fluid permeability is more homogenously distributed in all directions in the iliac crest. To the best of the authors’ knowledge, the comparison of the fluid permeability as a function of fluid flow direction and the comparison of different anatomical sites as presented within this study is the first of its kind, and might help explain the clinical observation that bone from the iliac crest has superior clinical outcome after intraoral transplantation when compared to bone from the alveolar crest. The regeneration of bone grafts depends on the revascularization of the graft, which is proposed to occur along the Haversian canals in autologous bone grafts (Nelson et al., 2006; Spin-Neto et al., 2014; Fretwurst et al., 2022). The flow dynamics of substances into the bone graft is predominantly from the residual bone of the recipient site, favoring an orientation of the vascular pore system of the graft in this direction (y-direction). In the cortical bone from the iliac crest, not only a higher permeability is found in the y-direction but also the permeability within the graft is higher due to the homogenous distribution of the vascular pore system in all directions. This difference in the architecture of the intracortical porosity might be responsible for the degree of osteoconductivity of an autologous bone graft. In addition to vascular porosity as a lineal dimension, the pore-network distribution and orientation could be an essential parameter for the design of bone graft substitutes (Feng et al., 2021). As this was the first study to implement fluid flow simulation in human biopsies, further larger sample number studies are needed to verify the data found within this study. In addition to increasing the number of samples, a more complex fluid simulation could be used if the composition of the vessel pores of bone transplants becomes known. Within this study, a simplified approach was used, that is, only a single-phase flow was considered, and soft tissue remnants inside the vessel pores were ignored. Furthermore, it should be noted that nonlinear effects of pore surfaces on fluid flow and permeability might play an important role in smaller pore diameters where surface–fluid interaction is more crucial than in larger pores.

## 5 Conclusion

Standard bone morphometric parameters (porosity and BV/TV) when using larger volume VOIs did not reveal a difference between the alveolar bone (AB) and cortical bone from the iliac crest [IC (CO)], whereas a significantly increased lacunar density in AB compared to IC (CO) was associated with a greater distance of mineralized tissue to the closest pore-vessel boundary in alveolar bone when compared to iliac bone. Assuming that the osteocyte network can contribute to mineral homeostasis through mineral exchange at their boundaries, we speculate that the increased number of osteocyte lacunae may compensate for the longer average distance of the mineralized bone tissue to the nearest vessel pore.

The spatial distribution of the intracortical pore system shows a multidirectional pattern in the cortical bone from the superior anterior iliac crest when compared to alveolar bone. Computational fluid simulations suggest that the average permeability is higher in IC than in AB, while at the same time, these calculations reveal a more pronounced anisotropy of the permeability in AB. The permeability in AB is pronounced in the z-axis and low in the y-axis. These differences in orientation, porosity, and permeability in the intracortical pore network architecture might be attributed to better osteoconductivity of the iliac bone when used as a bone graft in oral- and maxillofacial surgery and should be considered when designing bone substitute scaffolds.

## Data Availability

The original contributions presented in the study are included in the article/Supplementary Material; further inquiries can be directed to the corresponding authors.

## Abbreviations

3D, three-dimensional; AB, alveolar bone; BV, bone volume; CT, computed tomography; IC(CA), cancellous iliac crest; IC(CO), cortical iliac crest; ICS, Intracortical surface; IPS, intracortical pore system; LCN, osteocyte lacunar–canalicular system; LOA, level of alignment; N.Lc, number of lacunae; SA, surface area; SR µ-CT, synchrotron radiation micro-computed tomography; Tb.Sp, trabecular separation; Tb.Th, trabecular thickness; TV, total volume; VXL, voxel; VOI, volume of interest.
