# Supplementary material for: Comparison of the 3D-Microstructure Between Alveolar and Iliac Bone for Enhanced Bioinspired Bone Graft Substitutes
Source: Front Bioeng Biotechnol. 2022 Jun 17;10:862395. doi: 10.3389/fbioe.2022.862395 (PMC9248932; doi:10.3389/fbioe.2022.862395)
Supplement: Supplementary file 1 [file DataSheet1.docx]

Supplementary Material

# Supplementary Figures

| 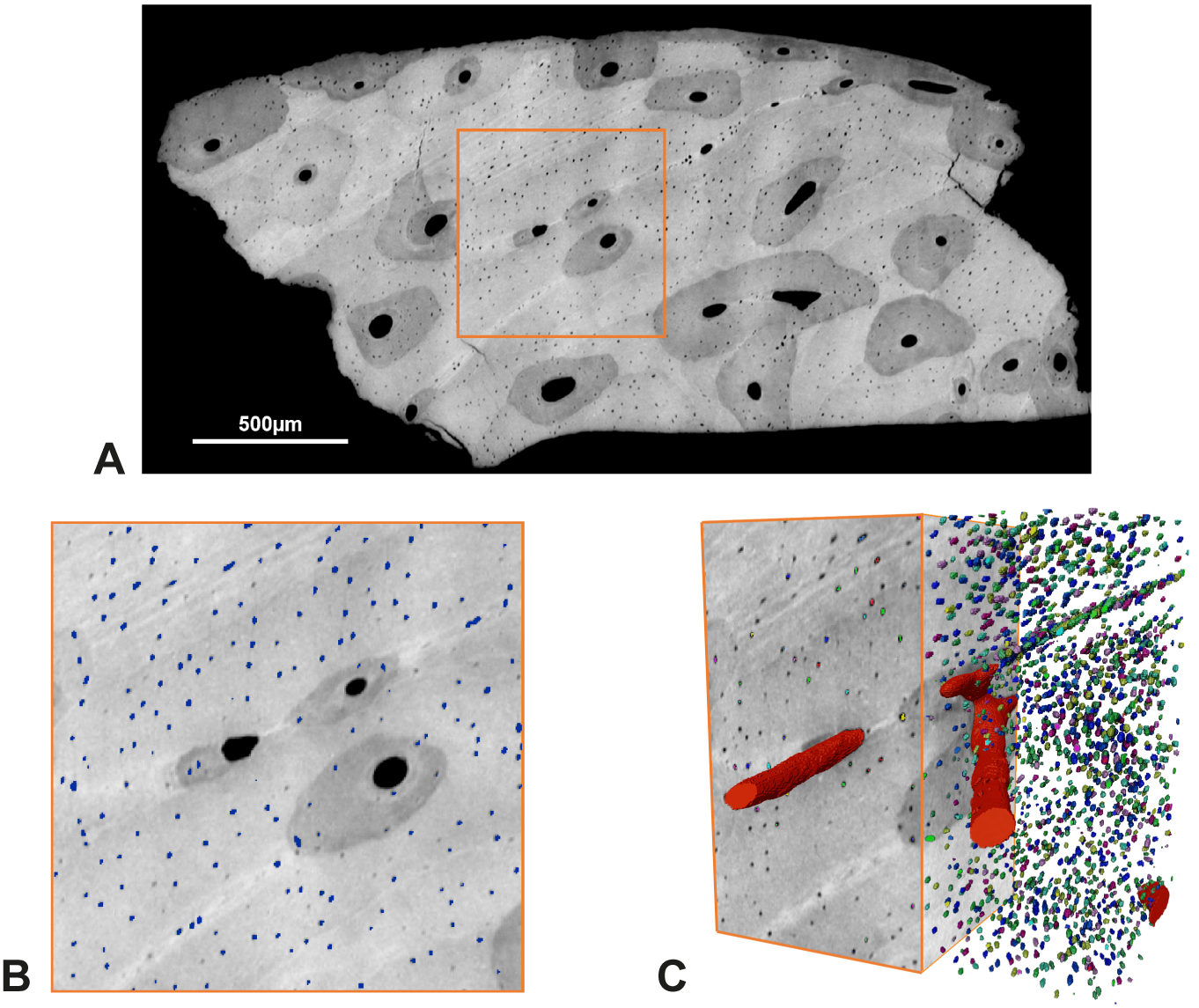 |
| --- |
| ***Supplementary Figure A1*:** Segmentation of the osteocyte lacunae in sample P8A.  ***A*** *Cross section of the complete dataset with the gray values representing different degrees of mineralization. Big pores are blood vessels, small pores are osteocyte lacunae. The small orange region is shown in* ***B****, where osteocyte lacunae are segmented and overlaid in blue. In* ***C*** *the orange cube is cropped such that the individually colored lacunae become visible. In this cube of ~0.17 mm3 around 4250 lacunae are detected. Blood vessels are rendered in red.* |

| 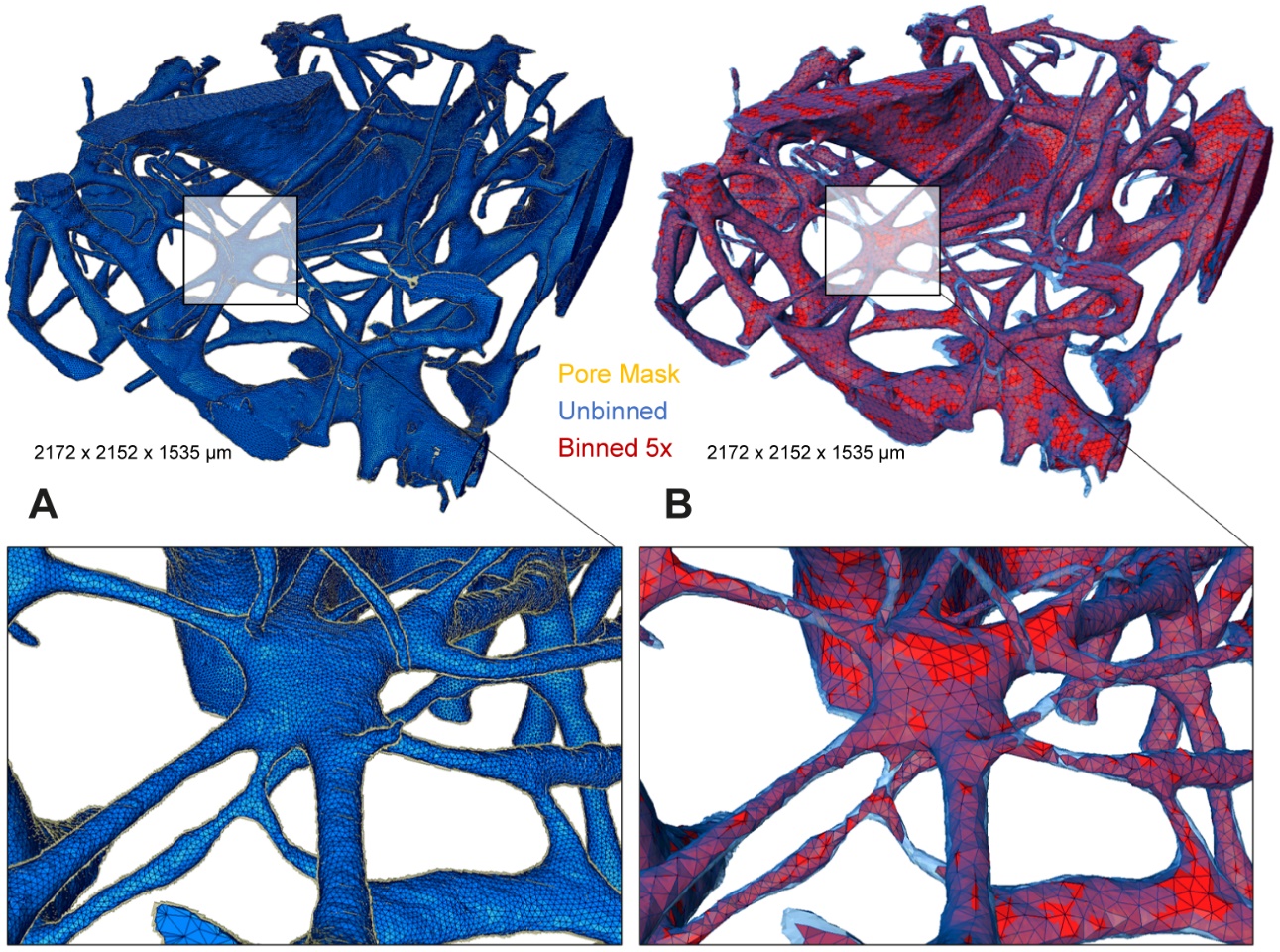 |
| --- |
| ***Supplementary Figure A2*:** Surfaces exemplarily generated for the pore spaces of sample P4A.  *In* ***A*** *the surface is generated directly on the complete, unbinned data. The original volume is overlayed in yellow. The zoom-in shows the good representation of the data since also small canals are visible.* ***B*** *shows a surface generated on a 5x binned dataset in red. The surface of the unbinned data is overlayed in blue. The zoom-in shows that small canals are lost due to the binning. The high resolution of the original data is thus needed for the surface generation.* |

| 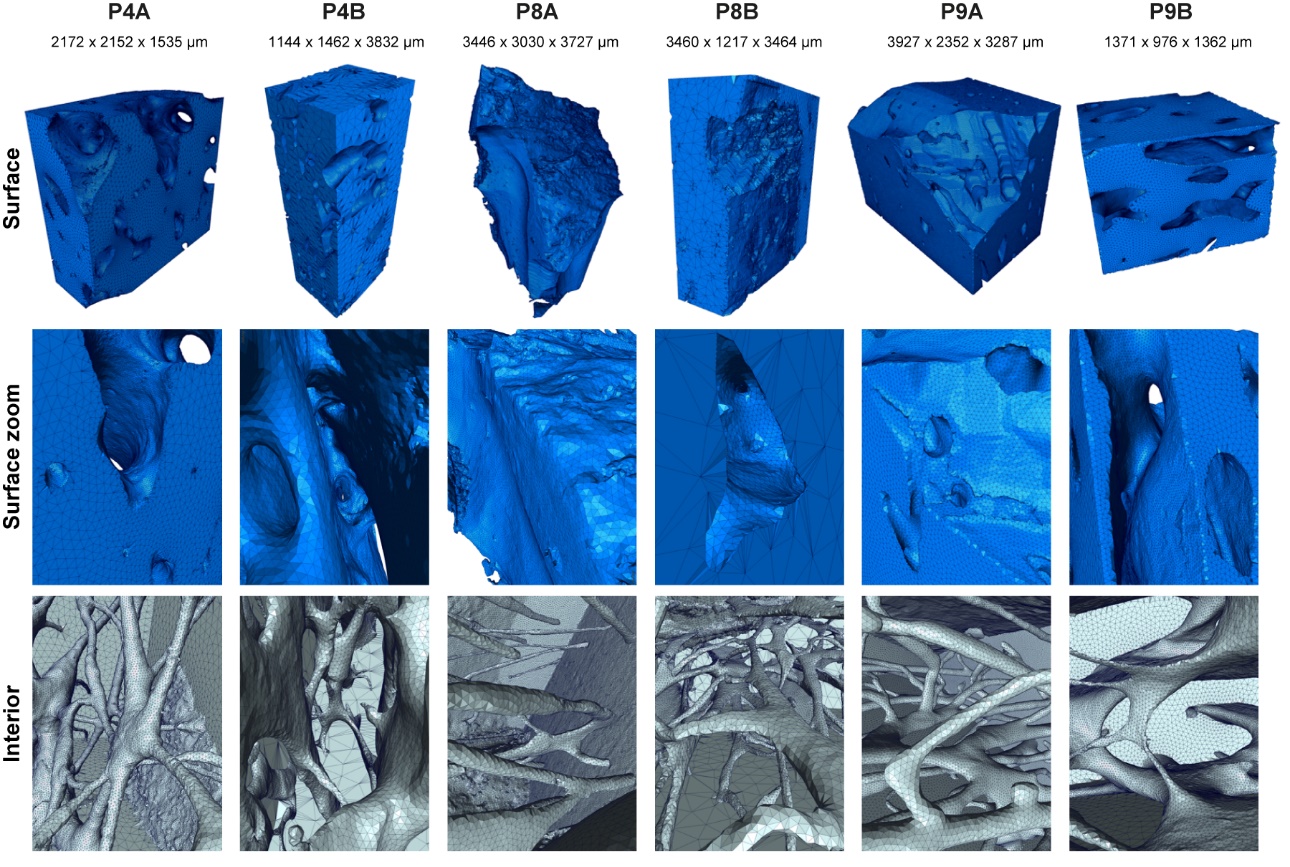 |
| --- |
| ***Supplementary Figure A3*:** Surfaces generated for all intensively characterized VOIs.  *The upper row shows the complete surface, the middle row shows zoom-ins at the surface and the bottom row shows an internal view of the surface. Thus the pore space becomes visible.* |

| 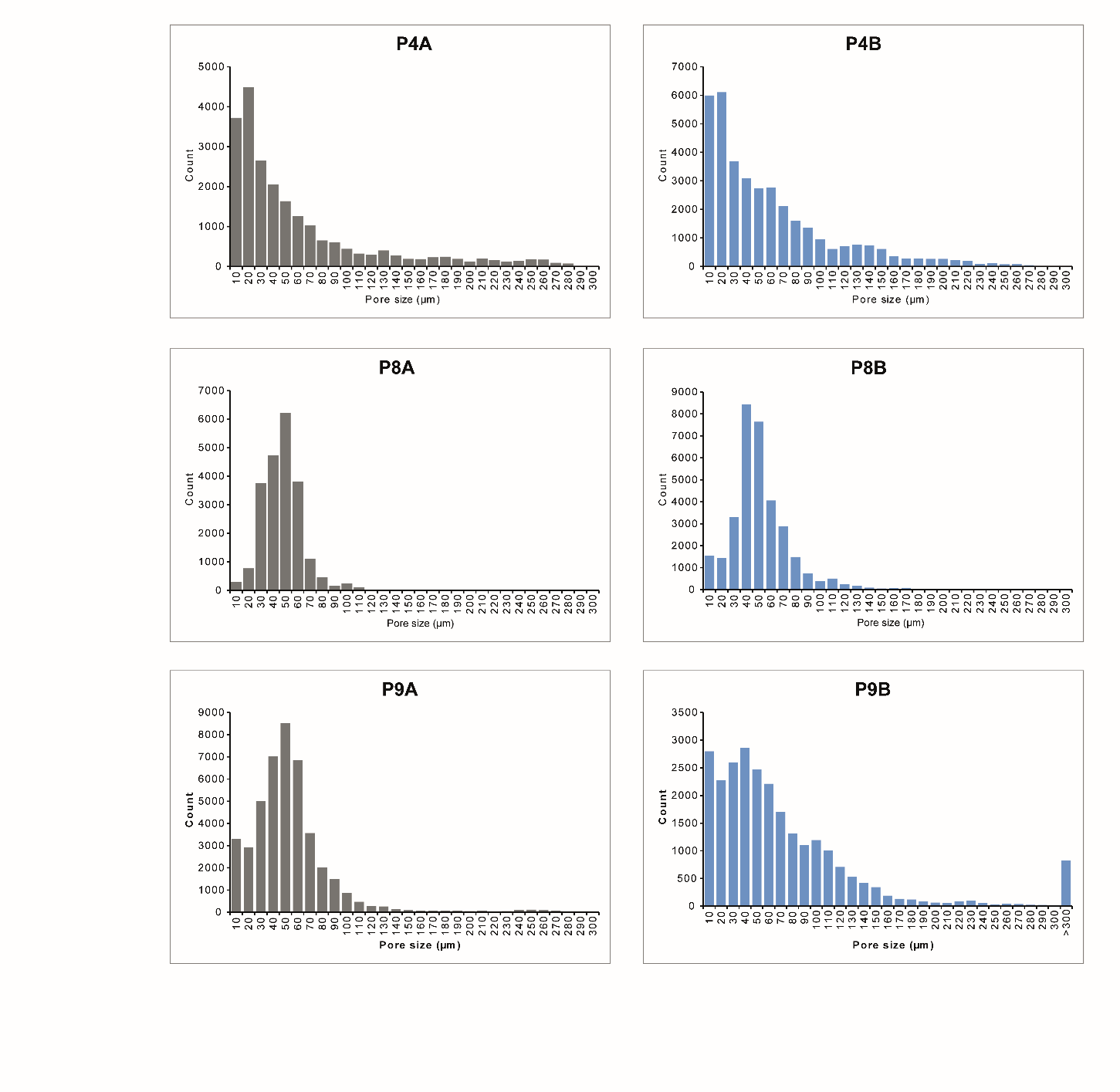 |
| --- |
| ***Supplementary Figure A4*:** Vascular pore size in samples from IC and AB.  *Histograms showing the distribution of pore sizes of the six samples studied in more detail in the IPS analysis.* |

# Supplementary Tables

| **Groups** | | **S.ID** | **TV (*mm³)*** | **BV (*mm³)*** | **SA (*mm^2^)*** | **BV/TV** | **Porosity (%)** | **SA/BV (*1/mm)*** | **N.Lc/BV** |
| --- | --- | --- | --- | --- | --- | --- | --- | --- | --- |
| AB | | 1 | 3.87 | 2.91 | 8.4 | 0.75 | 25.0 | 2.9 | 26892 |
|  |  | 2 | 0.97 | 0.69 | 3.7 | 0.71 | 29.1 | 5.3 | 23430 |
|  |  | 3 | 0.82 | 0.73 | 2.3 | 0.89 | 10.9 | 3.2 | 16168 |
|  |  | 4 | 1.75 | 1.30 | 5.5 | 0.74 | 26.0 | 4.3 | 29332 |
|  |  | 5 | 1.27 | 1.23 | 1.3 | 0.96 | 4.4 | 1.1 | 21503 |
|  |  | 6 | 1.74 | 1.05 | 7.3 | 0.60 | 40.0 | 7.0 | 24758 |
|  |  | 7 | 0.51 | 0.45 | 2.2 | 0.88 | 11.9 | 4.8 | 19911 |
|  |  | 8 | 0.94 | 0.91 | 1.5 | 0.97 | 2.9 | 1.7 | 23796 |
|  |  | 9 | 4.37 | 3.97 | 12.4 | 0.91 | 9.1 | 3.1 | 25097 |
|  |  | 10 | 37.78 | 9.42 | 109.4 | 0.25 | 75.1 | 11.6 | 23000 |
| Anterior iliac crest | IC(CO) | 1 | 3.56 | 2.98 | 13.6 | 0.84 | 16.1 | 4.6 | 19123 |
|  |  | 2 | 4.27 | 3.85 | 21.5 | 0.90 | 9.8 | 5.6 | 18978 |
|  |  | 3 | *NaN* | *NaN* | *NaN* | *NaN* | *NaN* | *NaN* | *NaN* |
|  |  | 4 | 5.79 | 4.83 | 30.5 | 0.83 | 16.6 | 6.3 | 14669 |
|  |  | 5 | 5.04 | 4.22 | 19.2 | 0.84 | 16.1 | 4.5 | 13939 |
|  |  | 6 | 0.45 | 0.38 | 1.9 | 0.86 | 14.3 | 5.0 | 14464 |
|  |  | 7 | 0.20 | 0.16 | 1.4 | 0.80 | 20.0 | 8.8 | 19050 |
|  |  | 8 | 1.99 | 1.78 | 8.4 | 0.89 | 10.7 | 4.7 | 12694 |
|  |  | 9 | 2.18 | 1.70 | 13.1 | 0.78 | 21.8 | 7.7 | 14290 |
|  |  | 10 | 2.01 | 1.66 | 10.9 | 0.83 | 17.2 | 6.5 | 26055 |
|  | IC(CA) | 1 | 14.28 | 3.33 | 30.6 | 0.23 | 76.7 | 9.2 | 15963 |
|  |  | 2 | *NaN* | *NaN* | *NaN* | *NaN* | *NaN* | *NaN* | *NaN* |
|  |  | 3 | 9.42 | 4.07 | 30.6 | 0.43 | 56.8 | 7.5 | 12515 |
|  |  | 4 | 20.42 | 1.24 | 16.0 | 0.06 | 93.9 | 12.9 | 16225 |
|  |  | 5 | 19.49 | 3.53 | 40.1 | 0.18 | 82.1 | 11.8 | 14984 |
|  |  | 6 | 10.86 | 2.45 | 29.4 | 0.23 | 77.4 | 12.0 | 14312 |
|  |  | 7 | 10.50 | 1.51 | 28.6 | 0.14 | 85.6 | 19.0 | 14545 |
|  |  | 8 | 3.60 | 1.25 | 12.3 | 0.35 | 65.3 | 9.8 | 11719 |
|  |  | 9 | *NaN* | *NaN* | *NaN* | *NaN* | *NaN* | *NaN* | *NaN* |
|  |  | 10 | 33.06 | 6.25 | 70.3 | 0.19 | 81.1 | 11.3 | 19958 |

***Supplemetary Table A1:*** Data set of bone morphometric parameters and osteocyte lacunae density.

*AB=alveolar bone; IC(CO)=cortical iliac crest; IC(CA)=cancellous iliac crest; S.ID=sample ID; TV=total volume; BV=bone volume; SA=surface area; N.Lc=number of lacunae; NaN=not a number.*

For gender comparison, the different skeletal sites were considered separately. For alveolar bone, cancellous iliac crest and cortical iliac crest, no significant differences between men and women were found concerning the studied parameters (BV/TV, porosity and N.Lc/BV). Nevertheless, on average, cortical iliac crest bone of women trended towards a higher porosity (mean: 18.24 %) than that of men (mean: 12.85 %; P = 0.063). (*Supplementary Table A2*).

| Profile | | | Mean based on | BV/TV | Porosity (%*)* | SA/BV *(1/mm)* | N.Lc./BV |
| --- | --- | --- | --- | --- | --- | --- | --- |
| AB | | Male (Ø 53.5 Years) | N = 5 | 0.78 ± 0.15 | 21.78 ± 14.82 | 4.30 ± 2.02 | 23497 ± 4729 |
|  |  | Female (Ø 60.0 Years) | N = 4*** | 0,88 ± 0.09 | 12.60 ± 8.83 | 2,98 ± 1.51 | 23351 ± 3206 |
|  |  | Mixed (Ø 56.3 Years) | N = 9 | 0. 82± 0.13 | 17.70 ± 12.74 | 3.71 ± 1.84 | 23432 ± 3878 |
| Anterior iliac crest | IC(CO) | Male (Ø 54.8 Years) | N = 4 | 0.87 ± 0.03 | 12.85 ± 3.17 | 5.40 ± 0.71 | 15201 ± 2669 |
|  |  | Female (Ø 60.0 Years) | N = 4* | 0.82 ± 0.03 | 18.50 ± 2.87 | 6.40 ± 2.18 | 16601 ± 2874 |
|  |  | Mixed (Ø 57.4 Years) | N = 8 | 0.84 ± 0.04 | 15.68 ± 4.12 | 5.90 ± 1.59 | 15901 ± 2675 |
|  | IC(CA) | Male (Ø 50.0 Years) | N = 4 | 0.27 ± 0.16 | 73.35 ± 16.10 | 10.55 ± 2,41 | 13693 ± 2007 |
|  |  | Female (Ø 58.3 Years) | N = 3* | 0.18 ± 0.05 | 81.47 ± 4.48 | 13.33 ± 5.08 | 15164 ± 726 |
|  |  | Mixed (Ø 54.2 Years) | N = 7 | 0.23 ± 0.13 | 76.83 ± 12.45 | 11.74 ± 3.70 | 14323 ± 1675 |

***Supplementary Table A2:*** Morphometric parameters profiles

*Data showing morphometric parameters subdivided into a “male”, “female” and “mixed” group. *=Sample 58 has been excluded as outlier. Values are given as mean ± standard deviation. AB=alveolar bone; IC(CO)=cortical iliac crest; IC(CA)=cancellous iliac crest;S.ID=sample ID; TV=total volume; BV=bone volume; SA=surface area; N.Lc=number of lacunae.*
